# Curb to Needle Time: A Five-Year Descriptive Analysis Evaluating a Mobile Stroke Unit in a Suburban EMS System

**DOI:** 10.5811/westjem.62948

**Published:** 2026-05-19

**Authors:** Eric Wetzel, Zachary Weisner, Marina Ulhaq, Matthew Kening, Alvin Wang, Gerald Wydro

**Affiliations:** Jefferson Northeast, Department of Emergency Medicine, Philadelphia, Pennsylvania

## Abstract

**Introduction:**

Stroke is a significant cause of mortality and long-term disability in the United States. Reduced time to thrombolytic therapy may lower stroke-related morbidity and mortality. The integration of a mobile stroke unit (MSU) into emergency medical service (EMS) systems of care has been focused in large metropolitan areas. A MSU may reduce the time from first medical contact (FMC) to thrombolytic therapy versus traditional care models, but this value must be evaluated in the context of the logistic and operational challenges of non-metropolitan EMS systems.

**Objective:**

To determine the impact of integrating an MSU into a large suburban EMS system with specific attention to key time metrics, logistics, and patient safety.

**Methods:**

We conducted a retrospective observational study of MSU dispatches by a County 911 emergency communication center (ECC) across a large suburban EMS system involving multiple agencies between August 1st, 2019, and July 31st, 2024. The MSU is a specialty ambulance with computerized tomography and telemedicine consultation capable of prehospital treatment of patients with thrombolytics. Criteria to dispatch the MSU were defined by the ECC caller-interrogation protocols for stroke and dispatcher discretion. Time metrics in minutes were reviewed for all incidents by the MSU, including dispatch to on-scene, and times from on-scene to key milestones such as imaging by computed tomography, neurology telemedicine consultation, thrombolytic therapy, and transport to the stroke center. Demographic data were obtained on those who received tPA and who were transported. Data are presented as a median with interquartile ranges (IQR).

**Results:**

Over a five-year period, the MSU had 1,752 dispatches, of which 717 patients were transported to the emergency department. The median time on scene before initiating transport to a comprehensive stroke center was 21 minutes (IQR 18–24). The median arrival to CT scan was 9 minutes (IQR 7 – 11), median FMC to neurology telemedicine consultation was 14 minutes (IQR 12 – 17), and dispatch to thrombolytic therapy was 38 min (IQR 31–43). A total of 91 patients (4%) received thrombolytics prehospital and FMC to thrombolytic therapy had a median time of 26 minutes (IQR 22 – 31). An equal number of men (47%) and women received thrombolytics (p value = 0.602) and the median age was 73. (IQR 64.5–83). The median time from reported last known well to thrombolytic was 71 minutes (IQR 53–136).

**Conclusion:**

We describe the successful integration of an MSU into a suburban EMS system involving multiple agencies with rapid FMC to thrombolytic administration time. Time metrics for each step to thrombolytic administration were consistent with little variance; no patient received thrombolytics beyond the 270 minutes safety limit. Of the 1,752 dispatches, 91 patients received thrombolytics. This should initiate further discussion on the clinical, financial, and operational impact of MSU care in suburban communities.

